# Canard analysis reveals why a large Ca^2+^ window current promotes early afterdepolarizations in cardiac myocytes

**DOI:** 10.1371/journal.pcbi.1008341

**Published:** 2020-11-04

**Authors:** Joshua Kimrey, Theodore Vo, Richard Bertram

**Affiliations:** 1 Department of Mathematics, Florida State University, Tallahassee, Florida, United States of America; 2 School of Mathematics, Monash University, Clayton, Victoria, Australia; 3 Department of Mathematics, and Programs in Neuroscience and Biophysics, Florida State University, Tallahassee, Florida, United States of America; King’s College London, UNITED KINGDOM

## Abstract

The pumping of blood through the heart is due to a wave of muscle contractions that are in turn due to a wave of electrical activity initiated at the sinoatrial node. At the cellular level, this wave of electrical activity corresponds to the sequential excitation of electrically coupled cardiac cells. Under some conditions, the normally-long action potentials of cardiac cells are extended even further by small oscillations called early afterdepolarizations (EADs) that can occur either during the plateau phase or repolarizing phase of the action potential. Hence, cellular EADs have been implicated as a driver of potentially lethal cardiac arrhythmias. One of the major determinants of cellular EAD production and repolarization failure is the size of the overlap region between Ca^2+^ channel activation and inactivation, called the window region. In this article, we interpret the role of the window region in terms of the fast-slow structure of a low-dimensional model for ventricular action potential generation. We demonstrate that the effects of manipulation of the size of the window region can be understood from the point of view of canard theory. We use canard theory to explain why enlarging the size of the window region elicits EADs and why shrinking the window region can eliminate them. We also use the canard mechanism to explain why some manipulations in the size of the window region have a stronger influence on cellular electrical behavior than others. This dynamical viewpoint gives predictive power that is beyond that of the biophysical explanation alone while also uncovering a common mechanism for phenomena observed in experiments on both atrial and ventricular cardiac cells.

## Author summary

EADs are pathological voltage fluctuations that can occur during the plateau or repolarizing phase of cardiac action potentials. The EADs of single cells, when embedded in a network of cardiac tissue, can lead to deadly cardiac arrhythmia. Because of this, many experimental and theoretical investigations have been conducted to uncover the biophysical and dynamical origins of EAD genesis. A recurring finding is that suitable changes in the properties of the inward L-type calcium current are sufficient for EAD production. A particularly important property of the L-type calcium current, with respect to EAD production, is the size of its window region. In this work, we use a novel geometric approach to analyze the role of the window region in cellular electrical dynamics using a low-dimensional ventricular action potential model. We illustrate the mechanism underlying window region-induced EADs, and demonstrate how the number of EADs produced can be predicted, using dynamical systems techniques together with canard theory. These techniques allow us to explain precisely why the model reproduces myriad experimental observations while also allowing us to make the testable predictions that either advancing the activation rate or slowing the inactivation rate of the L-type calcium current—changes that would reasonably be expected to increase its active duration and the likelihood of EADs—should, instead, reduce its active duration and the likelihood of EADs.

## Introduction

**Fig 1:**
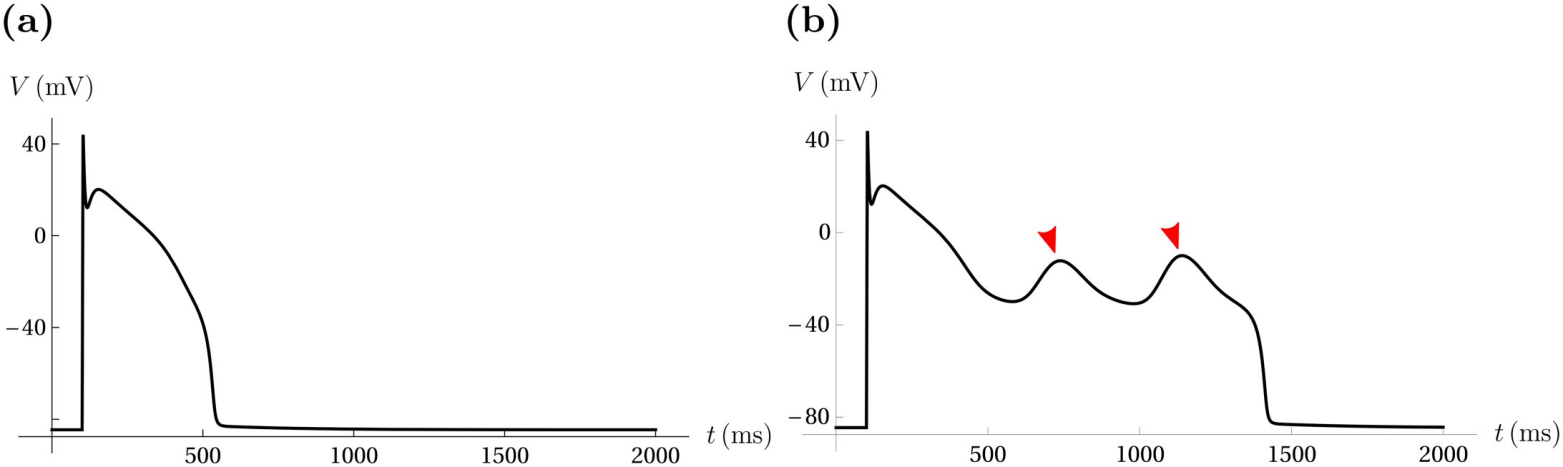
EADs in cardiac AP simulations. Cardiac APs can exhibit EADs in response to changes in the gating properties of L-type Ca^2+^ channels. (**a**) A simulated cardiac AP without EADs. (**b**) An AP exhibiting two EADs (red arrow markers) has a significantly prolonged duration.

**Fig 2:**
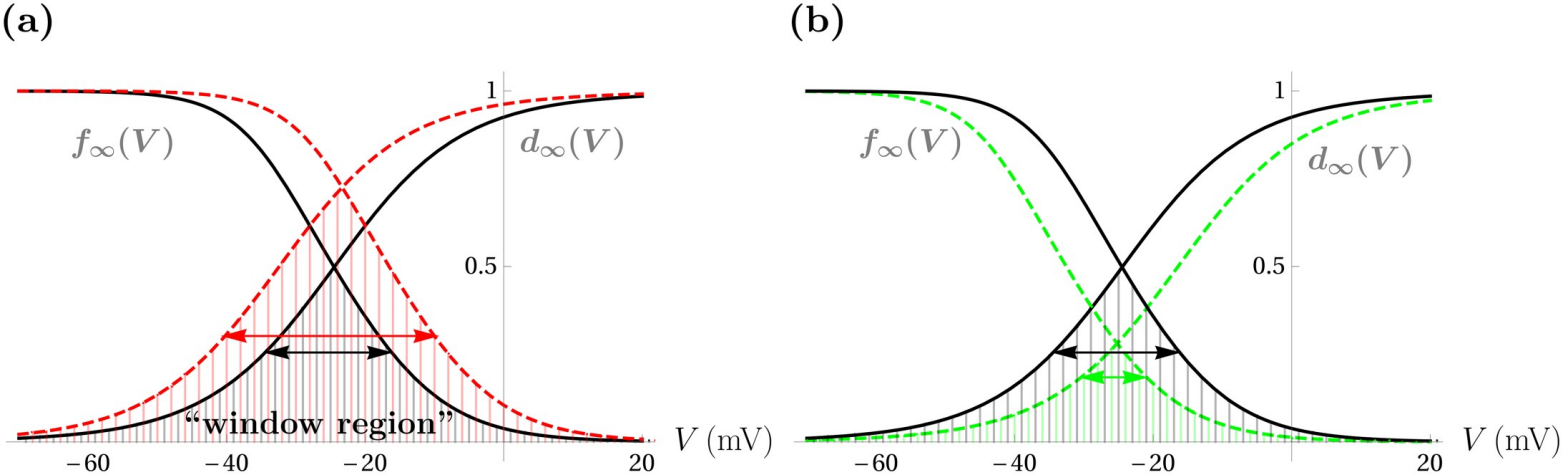
Schematic of symmetric broadening and narrowing of the *I*_Ca-L_ window region. (**a**) A left shift in *d*_∞_(*V*) and a right shift in *f*_∞_(*V*) (red curves) increases the area (filled with vertical red lines) under both curves as compared to the default setting (black curves with area colored with vertical gray lines). (**b**) A right shift in *d*_∞_(*V*) and a left shift in *f*_∞_(*V*) (green dashed curves) narrows the window region. The default area is colored gray and the reduced area is colored green.

Early afterdepolarizations (EADs) are pathological small oscillations in the membrane potential that can occur in the plateau or repolarization phase of cardiac action potentials (Fig 1b). These EADs prolong the action potential (AP) and can lead to arrhythmias such as tachycardia or fibrillation [1–5]. The origins of EADs and EAD-induced arrhythmia have been the focus of many experimental and theoretical studies which have been performed in isolated myocytes [4, 6–8] and in cardiac tissue [9–11], and much has been learned from these studies regarding the potential mechanisms underlying the abnormal electrical behavior. It is now clear that one mechanism for EADs is an abnormally broad “window region” in the L-type Ca^2+^ channels [9, 12, 13]. This window region is the range of voltages where the channel activation and inactivation curves overlap (Fig 2a). If this region is abnormally large, then the Ca^2+^ current remains active at plateau voltages and thereby contributes to the formation of EADs.

The importance of the *I*_Ca-L_ window current in EAD production was studied in a hybrid manner through the use of the dynamic clamp technique [14, 15]. This allows for the injection of an ionic current into a cell where the properties of the current are set using a mathematical model (see [16] for review). In the dynamic clamp studies of EADs, the L-type Ca^2+^ channels were blocked with nifedipine and then a model L-type Ca^2+^ current introduced using dynamic clamp. Using this approach, EADs evoked by H_2_O_2_ were recapitulated by simultaneously shifting both the activation and inactivation curve of model *I*_Ca-L_ to enlarge the window region [14]. In [15] it was shown that opening the window region by translating the model Ca^2+^ channel activation curve leftward and inactivation curve rightward by the same amount (termed a “symmetric opening” in [15]), reliably produced EADs in otherwise unaltered atrial rabbit and human cardiomyocytes. As was noted in both studies, these results are significant not only because they demonstrate the importance of the window region in EAD production, but also because with this approach the EADs are purely electrical. That is, they do not involve Ca^2+^-activated Ca^2+^ release (CICR) from the sarcoplasmic reticulum since there is no Ca^2+^ entry (the L-type channels have been blocked and the current introduced by dynamic clamp is carried by ions other than Ca^2+^). This is an important clarifying result, since it has been shown that EADs can be produced through CICR [17, 18], and using dynamic clamp as was done in [14, 15] allows one to focus in on the purely electrical EADs.

In addition to showing that symmetric opening of the window region facilitates EADs (and symmetric closing eliminates them), [15] showed that EADs could be produced by only translating the channel activation curve leftward or only translating the channel inactivation curve rightward (an “asymmetric opening” of the window). In fact, it was shown that translating the activation curve is more effective than translating the inactivation curve. The intuition behind this result is that it is more important for EAD production to activate the Ca^2+^ channels at low voltages than to keep them from inactivating at higher voltages.

It was also shown in [14] and [15] that narrowing the *I*_Ca-L_ window region can abolish pathological rhythms produced by experimental manipulations that leave the window region unchanged. In [14] it was shown that the EADs produced through hypokalemia, the reduction of the extracellular K^+^ concentration, [K^+^]_o_, could be abolished by narrowing the computer-generated *I*_Ca-L_ window region through either right shifts in the activation curve or left shifts in the inactivation curve. In [15], it was shown that EADs and non-repolarizing APs were produced by increasing the maximal conductance of computer-generated *I*_Ca-L_, and these rhythms could be countered by symmetric narrowing of the window region. These results make the point that the absolute size of the window region is not what matters; what matters is the size of the window in the context of other cellular parameters.

While the dynamic clamp studies together provide a systematic examination of how the size of the window region and the shifts of Ca^2+^ channel activation/inactivation curves affect purely electrical EADs, they provide no insight into the effects that these manipulations have on the dynamics underlying EADs. The electrical activity of cardiomyocytes is determined by nonlinear interactions of several ionic currents, described mathematically by nonlinear ordinary differential equations. Manipulating the window region changes parameters in these equations that cause EADs to occur, but how? Surely this is a generic property of the electrical system, since it has been demonstrated in both ventricular and atrial myocytes of both rabbits and humans. Because it is generic, it should be obtainable with low-dimensional models that include key ionic currents such as *I*_Ca-L_ and K^+^ current for repolarization of the AP. Such a model need not contain all the ionic currents found in myocytes, since these differ across species and between ventricular and atrial myocytes, yet the EAD behavior is produced by similar manipulations in each.

In this study, we determine why opening the window region facilitates EADs using a low dimensional model for a cardiac AP, consisting of four variables. After recapitulating the experimental results described above, we uncover the dynamic mechanism underlying these results. That is, we show why both symmetric and asymmetric opening of the window produces EADs, and we show why shifting the Ca^2+^ activation curve is more effective than shifting the inactivation curve. Finally, we show how and why changes in other parameters of the Ca^2+^ current, such as its maximal conductance and activation/inactivation time constants, affect EAD production. Our mathematical analyses (i) reproduce the results of dynamic clamp experiments and (ii) produce novel predictions that can be tested in future dynamic clamp experiments.

The mathematical analysis required to understand the EADs produced by the low-dimensional model is geometric singular perturbation analysis, also called fast-slow analysis (see [19] for review and [20] for a more extensive discussion). This takes advantage of a separation of timescales between those variables that change on a fast timescale (two variables in our case), and those that change on a much slower timescale (the other two variables). We used this model previously to demonstrate the dynamical mechanism of EADs [21], and we and others have used fast-slow analysis to analyze the dynamical basis of EADs with other low-dimensional models [22–26]. The particular model used affects the details of the phenomenon, some of which can be quite significant (e.g., whether APs are produced only through stimulation or produced intrinsically in a periodic fashion). However, the use of low-dimensional models rather than more biophysically accurate models [27–29] is motivated by the generic nature of the EAD behavior, and the fact that low-dimensional models can be analyzed much more effectively than high-dimensional models, as we demonstrate here.

## Methods

### The modified Luo-Rudy I model

The full Luo-Rudy I model [30] includes 6 voltage-dependent transmembrane ionic currents and a single variable accounting for the intracellular Ca^2+^ level. The inward currents include a spike-producing Na^+^ current (*I*_Na_), an L-type Ca^2+^ current (*I*_Ca-L_), and a constant conductance background current (*I*_b_). The outward currents include a delayed rectifier K^+^ current (*I*_K_), an extracellular [K^+^]-dependent K^+^ current (*I*_K1_), and a high-threshold K^+^ current (*I*_Kp_). Together, the Luo-Rudy I model contains 8 coupled nonlinear ordinary differential equations.

Our analysis, however, utilizes a reduced Luo-Rudy I model that only contains elements for the electrical component. This facilitates the mathematical analysis, and allows us to demonstrate that even a simple model can account for the findings of the dynamic clamp experiments [14, 15] that are the focus of this study. The modified model does not include equations for the intracellular Ca^2+^ concentration, because in the dynamic clamp experiments Ca^2+^ influx was pharmacologically blocked. Also, since the model Na^+^ current rapidly inactivates for *V* > −40 mV, i.e., *I*_Na_ ≈ 0 when EADs occur, this current is also excluded.

The modified model contains the following differential equations for the membrane electrical dynamics:

$$\begin{array}{c} \begin{array}{cc} C_{m}\frac{dV}{dt} & =-\left(I_{\text{Ca-L}}+I_{\text{K}}+I_{\text{K1}}+I_{\text{Kp}}+I_{\text{b}}\right)+I_{\text{stim}}\\ \frac{dd}{dt} & =\frac{d_{\infty }\left(V\right)-d}{\tau_{d}\left(V\right)}\\ \frac{df}{dt} & =\frac{f_{\infty }\left(V\right)-f}{\tau_{f}\left(V\right)}\\ \frac{dx}{dt} & =\frac{x_{\infty }\left(V\right)-x}{\tau_{x}\left(V\right)} \end{array} \end{array}$$ (1)

with ionic currents given by

$$\begin{array}{c} \begin{array}{cc} I_{\text{Ca-L}} & =g_{\text{Ca}}\;d\;f\;\left(V-V_{\text{Ca}}\right)\\ I_{\text{K}} & =g_{\text{K}}\;x\;{\text{X}}_{\text{i},\infty }\left(V\right)\;\left(V-V_{\text{K}}\right)\\ I_{\text{K1}} & =g_{\text{K1}}\;{\text{K}}_{\text{1},\infty }\left(V\right)\;\left(V-V_{\text{K1}}\right)\\ I_{\text{Kp}} & =g_{\text{Kp}}\;{\text{K}}_{\text{p},\infty }\left(V\right)\;\left(V-V_{\text{K1}}\right)\\ I_{\text{b}} & =g_{\text{b}}\;\left(V-V_{\text{b}}\right) \end{array} \end{array}$$ (2)

Here, *C*_*m*_ is membrane capacitance and *I*_stim_ is a time-dependent mollified square-wave stimulus current with amplitude 70 *μ*A/cm^2^ and 2 ms duration. Each transmembrane ionic current is formulated using the standard Hodgkin-Huxley formalism for excitable membranes [31, 32]. For example, in the expression for the Ca^2+^ current (*I*_Ca-L_), *g*_Ca_ is the maximal conductance, a parameter; the dynamic variables *d* and *f* are the open fraction of activation and inactivation gates, respectively, of all voltage-gated Ca^2+^ channels; and (*V* − *V*_Ca_) is the driving force for ion flux, where *V*_Ca_ is the reversal potential for Ca^2+^.

The *x* variable, which appears in the expression for *I*_K_, denotes the (slow) activation of this current. Each of the steady-state activation and inactivation functions, *j*_∞_(*V*) for *j* = *d*, *f*, *x*, X_1_, K_1_ and K_p_, are increasing and decreasing sigmoids, respectively. We use upper-case letters to denote quantities that adjust instantaneously to variation in *V* and thus remain at quasi-equilibrium. The time constants, *τ*_*d*_(*V*) and *τ*_*x*_(*V*), are bell-shaped, while *τ*_*f*_(*V*) is strictly increasing. The magnitudes of the time constants govern how quickly the companion gating variable adapts to changes in *V*. Small (large) values of *τ*_*j*_(*V*), *j* = *d*, *f*, *x* represent rapid (slow) adaptation. We refer the reader to [30] for the full model formulation.

All parameter values are identical to those used in [30], with the exception of the default maximal *I*_Ca-L_ conductance, *g*_Ca_, which is set at 0.112 mS/cm^2^ to facilitate EAD production. Some parameter values are varied to examine robustness of behaviors, and this is stated explicitly in the text of figures. Under all relevant parameter variations, the model (1) (absent *I*_stim_) possesses a stable equilibrium, *E*_1_, which functions as the cell rest state. Under parameter sets that are capable of producing EADs, (1) possesses two additional equilibria, *E*_2_ and *E*_3_, which are located at elevated membrane potentials. The equilibrium *E*_2_ can be either an unstable or stable spiral in parameter regions that produce APs with EADs, while *E*_3_ is always an unstable saddle point. The computer programs used to generate the results herein are available at: www.math.fsu.edu/∼bertram/software/cardiac.

### Model *I*_Ca-L_ and modifications of its “window region”

The manuscript focuses primarily on model responses to translations in the steady-state *I*_Ca-L_ activation and inactivation functions, *d*_∞_(*V*) and *f*_∞_(*V*), respectively. The region where these two curves overlap has been termed the “window region” [9] (see Fig 2a) and it has been implicated in the generation of EADs. Fig 2 shows plots of *d*_∞_(*V*) and *f*_∞_(*V*) under the default parameter set (black curves). In Fig 2a, the window region is increased by either (or both) translating *d*_∞_(*V*) leftward or translating *f*_∞_(*V*) rightward. In Fig 2b, the window region is reduced by translating *d*_∞_(*V*) rightward or translating *f*_∞_(*V*) leftward.

Both *d*_∞_(*V*) and *f*_∞_(*V*) are sigmoidal in *V*, and are parameterized by their steepness and by the value, *V*, of half-activation and half-inactivation, respectively. Translation of each curve is accomplished by varying its half-activation/inactivation value. For clarity and consistency with experimental works, we discuss variation in the half-activation/inactivation values of the curves with reference to the default parameter set and denote the direction and magnitude of variation in the half-activation value of *d*_∞_(*V*), for instance, by Δ*V*_1/2_(*d*_∞_). We similarly denote translations in *f*_∞_(*V*) by Δ*V*_1/2_(*f*_∞_). We also note that the enlargement of the window region in Fig 2a and the narrowing of the window region in Fig 2b are symmetric with respect to the direction and magnitude of the translation in each curve. That is, the translations of both curves in each panel are equal in magnitude, but opposite in sign (i.e., for Fig 2a, Δ*V*_1/2_(*f*_∞_) = -Δ*V*_1/2_(*d*_∞_)).

## Results

### Symmetric enlargement of the model window region can produce EADs

Previous experimental and mathematical studies of EADs have concluded that most EADs occur while voltage is within the interval where the activation and inactivation curves (*d*_∞_(*V*)and *f*_∞_(*V*), respectively, in our model) of *I*_Ca-L_ overlap, termed the “window region”. The experimental work [15] showed that symmetric enlargement of the window region can lead to EADs as well as the inability of the cell to repolarize (see Fig 5 of [15]) in response to low-frequency periodic pacing.

**Fig 3:**
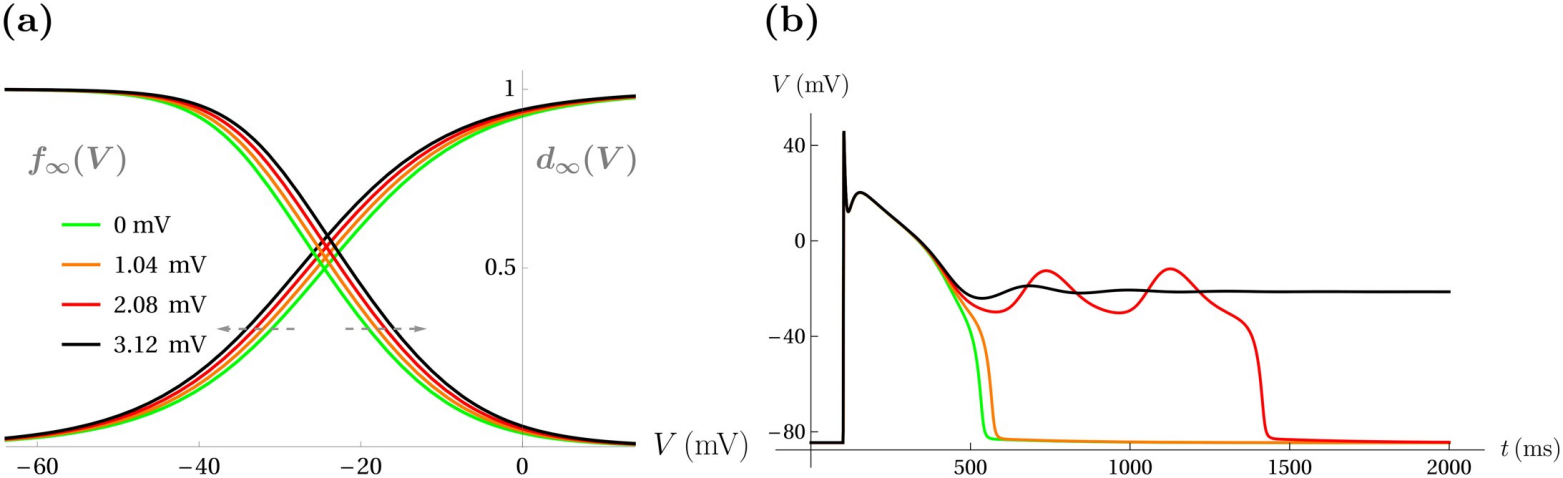
A sufficiently large symmetric broadening of the window region can lead to EADs and repolarization failure in response to a stimulus pulse. (**a**) An equally-spaced sequence of three color-coded symmetric window broadening translations in *d*_∞_(*V*) and *f*_∞_(*V*) (orange, red, and black curves) are shown alongside the default curves *d*_∞_(*V*) and *f*_∞_(*V*) (green). The magnitudes of each of the simultaneous changes to both Δ*V*_1/2_(*d*_∞_) and Δ*V*_1/2_(*f*_∞_) are shown in the legend. (**b**) The color-matched model responses correspond to the manipulations in panel (a).

Representative responses of the model cell to symmetric broadening of the *I*_Ca-L_ window region are shown in Fig 3. Fig 3a shows a sequence of symmetric translations of both the steady-state activation and inactivation curves, which enlarge the window region. The green curves denote the default state of the model window region (Δ*V*_1/2_(*d*_∞_) = Δ*V*_1/2_(*f*_∞_) = 0 mV), while the black curves denote the largest translation depicted (Δ*V*_1/2_(*d*_∞_) = -3.12 mV and Δ*V*_1/2_(*f*_∞_) = +3.12 mV). Fig 3b shows color-coded voltage traces of the corresponding model responses to a single stimulus pulse under each translation condition from Fig 3a. The green voltage trace shows the standard cardiac action potential without alteration. The orange trace shows a slightly prolonged action potential in response to a small symmetric enlargement of the window (Δ*V*_1/2_ = 1.04 mV), but no EADs. The red trace shows that a larger translation (Δ*V*_1/2_ = 2.08 mV) elicits two EADs, which prolong the duration of the action potential dramatically. Finally, the black trace shows that a sufficiently large increase in the size of the window region (Δ*V*_1/2_ = 3.12 mV) leads to repolarization failure, where the cell remains at a depolarized voltage.

### Left shifts in the activation curve are more effective at facilitating EADs than right shifts in the inactivation curve

Using the dynamic clamp technique to inject a model Ca^2+^ current into a cardiomyocyte, it was shown that simultaneous broadening of the window region by shifting both the Ca^2+^ current activation and inactivation curves facilitates EAD production and repolarization failure [14, 15]. Translations in either the activation or inactivation curves, but not both, were also examined. It was determined that left-translations in the activation curve alone were a more potent driver of EADs and repolarization failure than right-translations in the inactivation curve alone [15]. That is, using equal-in-magnitude translations of each curve in separate trials, left-translations in *d*_∞_(*V*) more often led to EADs and repolarization failure than did right-translations of *f*_∞_(*V*).

**Fig 4:**
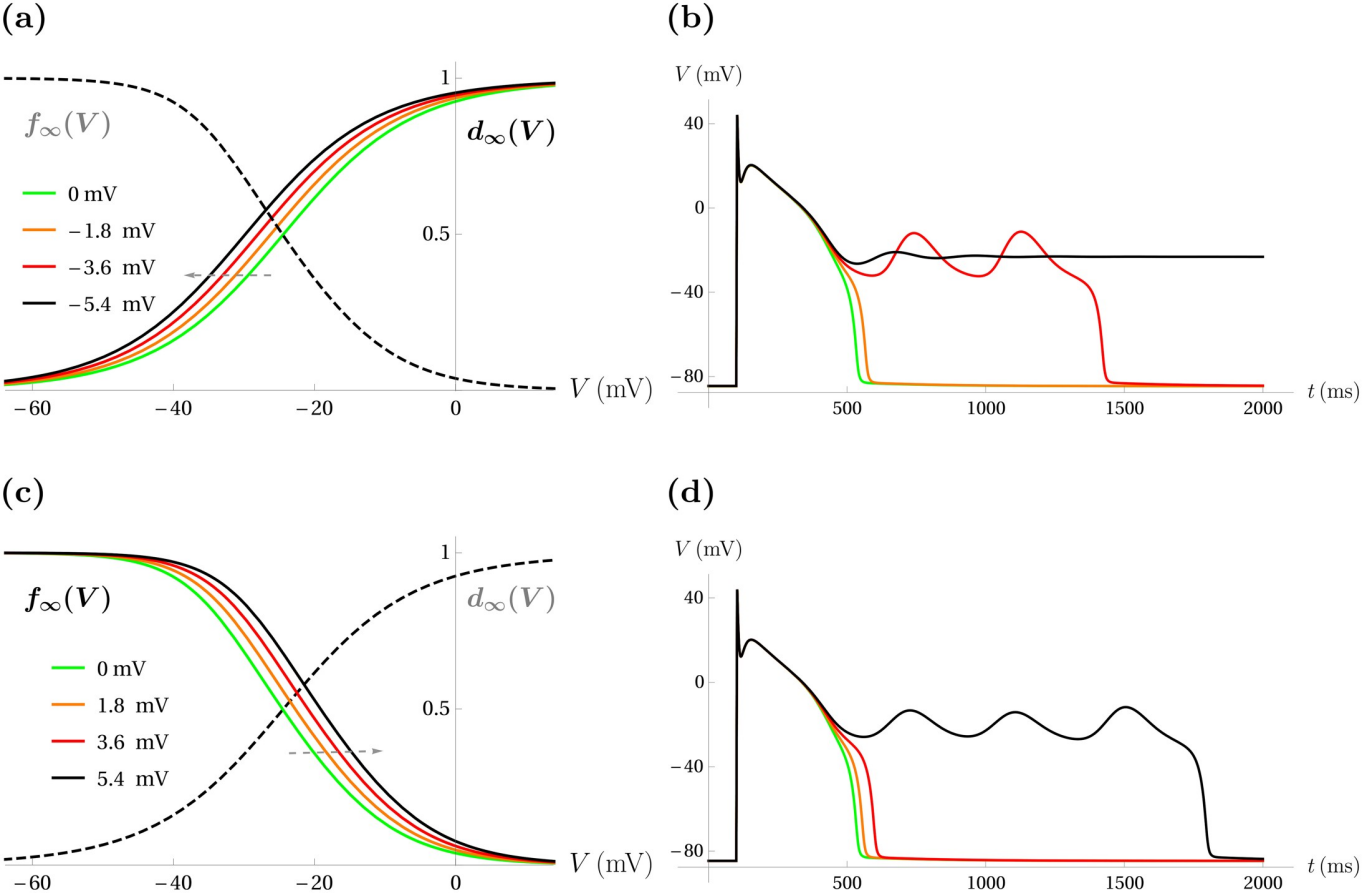
Left shifts in the Ca^2+^ current activation curve are more effective at inducing EADs and repolarization block than right shifts in the inactivation curve. (**a**) Three equally-spaced left shifts in *d*_∞_(*V*) (ordered orange, red, then black) are shown, while leaving *f*_∞_(*V*) (dashed, black) unchanged. As in Fig 3, green denotes the default. The shifts are given in the legend. (**b**) The model responses to the left-translations shown in (a) mirror those of Fig 3b: sufficiently large translation induces two EADs (red trace) and the largest translations lead to repolarization failure (black trace). (**c**) Right shifts in *f*_∞_(*V*) of equal size to those of (a). (**d**) The model responses to increasing Δ*V*_1/2_(*f*_∞_) are less severe than those of equally-sized changes in Δ*V*_1/2_(*d*_∞_): the largest change in Δ*V*_1/2_(*f*_∞_) produces EADs (black trace) instead of repolarization failure.

To test this experimental finding with the modified Luo-Rudy model, we first applied left-shifts of the Ca^2+^ activation curve, *d*_∞_(*V*), of magnitudes such that the first shift (Δ*V*_1/2_(*d*_∞_) = −1.8 mV) resulted in a longer action potential, the second (Δ*V*_1/2_(*d*_∞_) = −3.6 mV) resulted in an action potential with two EADs, and the third shift (Δ*V*_1/2_(*d*_∞_) = −5.4 mV) resulted in repolarization failure. That is, the magnitude of the shifts were chosen so that the responses mimicked those of Fig 3. These are shown in Fig 4a and 4b. We then applied right shifts of the same magnitude to the Ca^2+^ inactivation curve, *f*_∞_(*V*). These translations and the responses are shown in Fig 4c and 4d. In this case, EADs are only produced with the largest translation (Δ*V*_1/2_(*f*_∞_) = 5.4 mV), and none of the translations result in repolarization failure. Thus, the left shifts in *d*_∞_(*V*) are more potent than equal right shifts in *f*_∞_(*V*) at evoking EADs and repolarizaiton failure, as was shown experimentally in [15].

### Enlarging the model window region generically leads to EADs and repolarization failure

**Fig 5:**
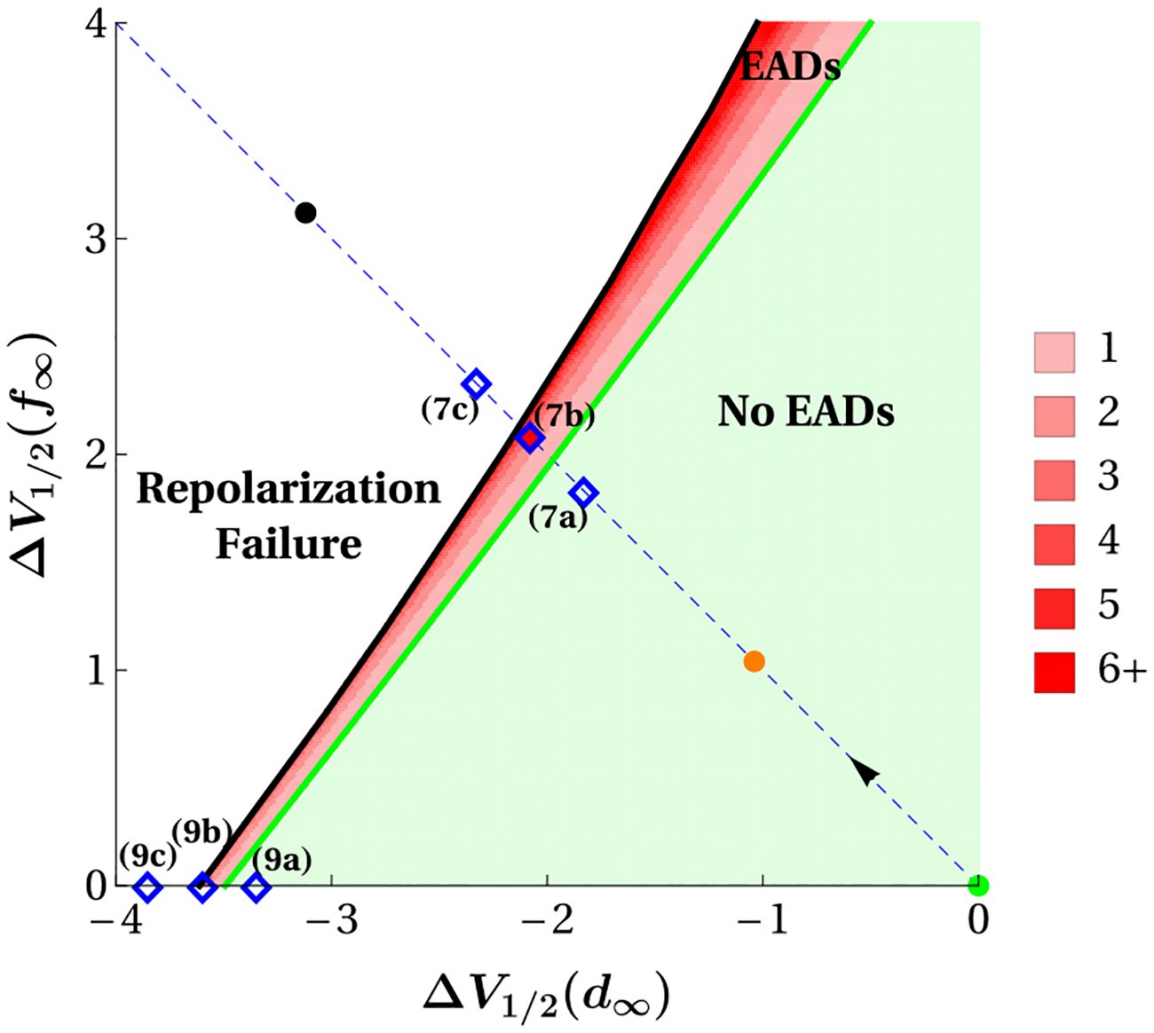
Model responses to a single depolarizing pulse over a uniform grid in the (Δ*V*_1/2_(*d*_∞_), Δ*V*_1/2_(*f*_∞_)) parameter plane (units in mV). The green region, labelled “No EADs”, denotes solutions that do not exhibit EADs before returning to rest. The white region, labelled “Repolarization Failure”, denotes solutions that can exhibit EADs around an elevated membrane potential, but remain depolarized. The red region, labelled “EADs”, contains solutions that exhibit EADs and return to rest at the end of the action potential. Darker shades of red in this region denote increasing numbers of EADs in response to the pulse. The dashed blue line segment gives the path in parameter space that corresponds to symmetric window-broadening. Green, red, orange, and black disks along this path correspond to the specific parameter values that produce the color-matched window regions and model responses shown in Fig 3. Blue ⋄ markers labeled 7a, 7b, 7c and 9a, 9b, 9c are parameter sets whose solutions are viewed in (*f*, *x*, *V*) phase space in Figs 7 and 9, respectively. The slope (>1) of the green curve, which marks the boundary between the “No EADs” and “EADs” regions, explains why left shifts in *d*_∞_(*V*) are a more reliable source of EAD production than right shifts in *f*_∞_(*V*).

In this section, we quantify the effectiveness of activation/inactivation curve shifts in inducing pathological behavior by examining combinations of the shifts, Δ*V*_1/2_(*d*_∞_) and Δ*V*_1/2_(*f*_∞_), that produce EADs or repolarization failure. This is organized using a two-dimensional grid in Δ*V*_1/2_(*d*_∞_) and Δ*V*_1/2_(*f*_∞_), noting that left-shifts in *d*_∞_(*V*) induce EADs, while right-shifts in *f*_∞_(*V*) induce EADs. Moving leftward along the Δ*V*_1/2_(*d*_∞_)-axis (to negative values) in Fig 5 corresponds to left shifts in *d*_∞_(*V*), while moving upward along the Δ*V*_1/2_(*f*_∞_)-axis (to positive values) corresponds to right shifts in *f*_∞_(*V*). To determine model behavior at each point in the 300 × 300 grid of parameter values, the model was integrated for 10,000 ms at each point using the stable rest state as initial condition. In each case, a supra-threshold pulse of current of amplitude 70 *μ*A/cm^2^ was applied for 2 ms to initiate an AP.

The light green region in Fig 5, labeled “No EADs”, shows parameter values that produce action potentials without EADs. These solutions may, however, exhibit prolonged action potentials (e.g., orange trace, Fig 4b). The white region, labeled “Repolarization Failure”, denotes the region of parameter combinations that produce solutions that remain in the depolarized state in response to the stimulus pulse (e.g., black trace, Fig 4b). The red region denotes those parameter combinations that produce solutions that contain EADs, but return to rest following the pulse (e.g., red trace, Fig 4b). A dashed curve is superimposed on the figure denoting the path in the (Δ*V*_1/2_(*d*_∞_), Δ*V*_1/2_(*f*_∞_))-plane used to produce Fig 3. The sequence of parameter sets shown in Fig 3 are marked with color-matched disks: the green disk signifies the default parameter set, the red disk (within the blue “⋄” marker labeled “(b)”) lies within the “EADs” region, and the black disk lies in the “Repolarization Failure” region.

The red “EADs” region possesses finer structure than the light green or white regions. Increasingly darker shades of red are used to indicate incremental increases in the number of EADs produced: 6 or more EADs are produced within the darkest shade of red, and some parameter combinations in this region produce solutions with as many as 40 EADs. The diagram shows that variation in the number of EADs elicited in this red region is organized into bands that gradate the transition from “No EADs” to “Repolarization Failure” and that the size of the bands declines corresponding to more EADs. That is, the red “EADs” region is dominated by solutions exhibiting few, rather than many, EADs. This finding predicts that action potentials with relatively few EADs should be more readily observed in experimental settings, as does indeed seem to be the case in published voltage traces from isolated myocytes [7, 14, 15].

The finding (both in the model and experimentally) that EADs are produced more effectively by left shifts in *d*_∞_(*V*) than right shifts in *f*_∞_(*V*) is evident in Fig 5. The curve that separates the “No EADs” region from the “EADs” region (green line) is approximately linear with slope *s* ≈ 1.34. Because the slope is greater than 1, it takes a larger change in Δ*V*_1/2_(*f*_∞_) than in Δ*V*_1/2_(*d*_∞_) to move from a parameter combination producing a pure action potential to one producing an action potential with EADs.

We can also use the slope of the green EAD boundary curve to make predictions about the potential therapeutic effects of window-shrinking shifts in either *d*_∞_(*V*) or *f*_∞_(*V*). Because the slope is greater than 1, the horizontal (rightward) distance from any point in either the “EADs” (red) or “Repolarization Failure” (white) regions to the green boundary between the “EADs” and “No EADs” regions is always smaller than the vertical (downward) distance. Thus, small window-shrinking translations in *d*_∞_(*V*) should be a more reliable therapeutic target than small window-shrinking translations of *f*_∞_(*V*) for the elimination of pathological rhythms (EADs or repolarization failure) induced by an enlarged window region.

An additional feature of the diagram that would not be readily discernible from either experiments or simulations is that the “EADs” region (bounded between the green and black curves) grows in width for increasing values of Δ*V*_1/2_(*f*_∞_) but, shrinks in width for decreasing values of Δ*V*_1/2_(*d*_∞_), even though both of these manipulations enlarge the window region. This feature of the diagram arises from the fact that the slope of the (almost linear) black curve, marking the boundary between the “EADs” and “Repolarization Failure” regions, has an even larger average slope than that of the green boundary curve. This feature of the grid makes the experimentally testable prediction that the transition of a cell from EADs to repolarization failure should also occur for smaller window-enlarging shifts in *d*_∞_(*V*) than *f*_∞_(*V*). That is, given a cell exhibiting EADs due to an enlarged window region, small increases in the magnitude of Δ*V*_1/2_(*d*_∞_) should be more likely to lead to repolarization failure than small increases in Δ*V*_1/2_(*f*_∞_). In addition, this predicted disparity between the effects of Δ*V*_1/2_(*d*_∞_) and Δ*V*_1/2_(*f*_∞_) in producing repolarization failure should be more pronounced than the disparity observed for the production of EADs shown in Fig 4.

### Fast-slow analysis reveals a mechanism for EAD generation

We have seen that broadening the *I*_Ca-L_ window region can lead to EADs and repolarization failure. Here we explore why, using a fast-slow analysis. Fast-slow analysis splits a model into (simpler) lower-dimensional subsystems in order to analyze these subsystems semi-independently and stitch together the results. In [21], we showed that (1) possesses a multi-timescale structure. This structure is reflected by the rapid upstrokes and downstrokes of the AP, with long depolarized plateau (Fig 1b). Specifically, we showed that the 4-dimensional model contains fast variables *V* and *d* (voltage and *I*_Ca-L_ activation), and slow variables *f* and *x* (*I*_Ca-L_ inactivation and *I*_K_ activation). The parameter *C*_*m*_ approximately characterizes the timescale separation, with *C*_*m*_ → 0 (termed the *singular limit*) yielding the decomposition of (1) into separate fast and slow subsystems (see [21] for details).

**Fig 6:**
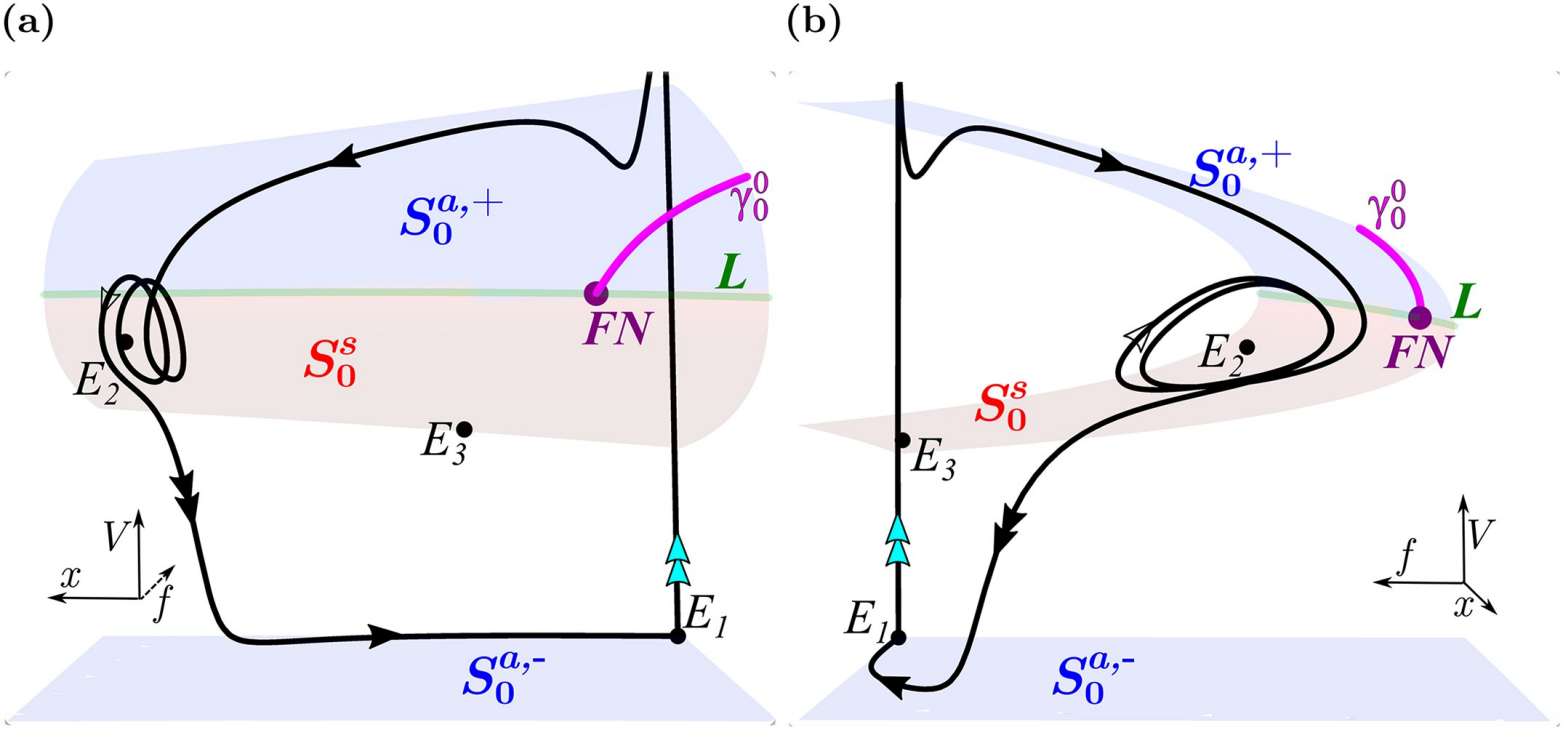
Two views of the EAD-containing voltage trace from Fig 3b superimposed on the critical manifold in (*f*, *x*, *V*) phase space. (**a**) (*x*, *V*)-dominant view. (**b**) (*f*, *V*)-dominant view. The superimposed solution (black) is comprised of: 1) a fast upstroke (cyan double arrows) caused by a stimulus pulse applied at rest (stable equilibrium *E*_1_), 2) slow evolution (single arrow) along the upper attracting sheet of the critical manifold, $S_{0}^{a,+}$ (upper blue surface), during the plateau phase, 3) oscillatory EADs (unfilled arrows) near the fold curve, *L* (green), 4) fast transition (double arrows) toward the lower attracting sheet, $S_{0}^{a,-}$, and 5) slow return (single arrow) to *E*_1_ along $S_{0}^{a,-}$. The folded node singularity (*FN*, purple marker), a pseudo-equilibrium of the slow subsystem, lies within *L*; its associated singular strong canard, $\gamma_{0}^{0}$ (magenta), a special solution of the slow subsystem, together with *L*, bounds the region of solutions of the slow subsystem, that are funneled through the folded node. Parameter values: Δ*V*_1/2_(*d*_∞_) = -Δ*V*_1/2_(*f*_∞_) = -2.08 mV.

With our (2,2)–fast-slow splitting, the 2-dimensional *fast subsystem*

$$\begin{array}{c} \begin{array}{cc} C_{m}\frac{dV}{dt} & =-(I_{\text{Ca-L}}+I_{\text{K}}+I_{\text{K1}}+I_{\text{Kp}}+I_{\text{b}})\\ \frac{dd}{dt} & =\frac{d_{\infty }\left(V\right)-d}{\tau_{d}\left(V\right)}\\ \frac{df}{dt} & =0\\ \frac{dx}{dt} & =0 \end{array} \end{array}$$ (3)

is an approximation of the fast motions of (1) (see Fig 6, double arrows) in which the slow variables, *f*, and *x*, are treated as parameters. The time-dependent forcing, *I*_stim_, is dropped from the *V*-equation because *I*_stim_ ≈ 0 after the stimulus has been applied. The equilibria of (3) (traced out by independent variation in *f* and *x*) form a 2-dimensional surface, called the *critical manifold*. Fig 6 shows two views of the EAD-containing voltage trace from Fig 3b in (*f*, *x*, *V*) phase space and superimposed on the critical manifold. The critical manifold is comprised of attracting ($S_{0}^{a,+}$ and $S_{0}^{a,-}$, blue) and saddle-type ($S_{0}^{s}$, red) sheets that are connected by curves of fold points. Only the upper fold, *L* (green), falls within the physiologically relevant domain (the lower curve is out of the frame of the figure, so not visible). The stability properties of the critical manifold are determined by linear stability analysis of the fast subsystem. The true equilibria, *E*_1_, *E*_2_, and *E*_3_ of the full system (1) persist as equilibria of the fast subsystem (3). While *E*_2_, under this parameter set, is a stable spiral of the full flow (1) (i.e., for *C*_*m*_ = 1 *μ*F/cm^2^), it becomes a saddle point (located on $S_{0}^{s}$) of the fast subsystem (3) (i.e., for *C*_*m*_ = 0 *μ*F/cm^2^). We note that there are no Hopf bifurcations in the fast subsystem, so EADs do not arise as oscillations in the fast subsystem as they do in previous works (e.g., [22]).

The 2-dimensional *slow subsystem*

$$\begin{array}{c} \begin{array}{cc} 0 & =-(I_{\text{Ca-L}}+I_{\text{K}}+I_{\text{K1}}+I_{\text{Kp}}+I_{\text{b}})\\ 0 & =\frac{d_{\infty }\left(V\right)-d}{\tau_{d}\left(V\right)}\\ \frac{df}{dt} & =\frac{f_{\infty }\left(V\right)-f}{\tau_{f}\left(V\right)}\\ \frac{dx}{dt} & =\frac{x_{\infty }\left(V\right)-x}{\tau_{x}\left(V\right)} \end{array} \end{array}$$ (4)

is an approximation of the slow motions of (1) (see Fig 6, solid single arrows) in which *V* and *d* are assumed to be at quasi-equilibrium. Hence, solutions of the slow subsystem (4) are slaved to the critical manifold.

To understand the trajectory of the full model (1), one can concatenate orbit segments from the fast and slow subsystems. This is only an approximation, however, and as we see below neither the fast nor the slow dynamics independently explain the EADs. The fast and slow motions are denoted using single and double arrows, respectively. A sufficiently strong stimulus pulse applied to the rest state, *E*_1_(on $S_{0}^{a,-}$), triggers a rapid excursion toward $S_{0}^{a,+}$ (cyan double arrows denote that this motion is the result of a depolarizing pulse). Once near $S_{0}^{a,+}$, the solution moves slowly as it follows $S_{0}^{a,+}$ closely during the plateau phase, toward the fold, *L*. The oscillations that occur near *L* are the EADs. Once several of these have occurred, the trajectory moves rapidly toward $S_{0}^{a,-}$. It then follows $S_{0}^{a,-}$ closely as it moves slowly back towards the rest state, *E*_1_.

The unfilled arrows along the oscillatory EAD portion of the solution indicate that this motion is neither strictly fast nor slow. Indeed, it is precisely at the fold curve *L* where the fast-slow approximation breaks down. That is, the fold marks the transition boundary between the non-overlapping regions of validity for the fast and slow subsystem approximations.

Without a fast subsystem mechanism for the generation of EADs, we turn to further inspection of the slow subsystem. The general procedure for this analysis can be found in the review article [33] and the details for the particular case of the slow subsystem (4) can be found in [21]. Here, we summarize the key elements. Solutions of the slow subsystem, when initiated on $S_{0}^{a,+}$, flow toward the fold curve. Upon reaching the fold, these solutions typically transition to the fast subsystem dynamics, so the trajectory quickly moves from the top sheet $S_{0}^{a,+}$ to the bottom sheet $S_{0}^{a,-}$. However, there may exist distinguished points on the fold curve called *folded node singularities* [34] (Fig 6; purple marker, “*FN*”) at which solutions can cross from $S_{0}^{a,+}$ to $S_{0}^{s}$, remain governed by the slow subsystem dynamics, and follow $S_{0}^{s}$ for long times. Such solutions are known as *singular canards*. Given the presence of a folded node singularity, there is a special singular canard that acts as a boundary along $S_{0}^{a,+}$ between solutions that, upon reaching the fold, either funnel through to the folded node or transition to the fast dynamics. This special singular canard is called the *singular strong canard* (Fig 6; $\gamma_{0}^{0}$, magenta).

For *C*_*m*_ > 0, singular canards become solutions of the full model (1) with similar properties, i.e., they remain near $S_{0}^{s}$ for long times on the slow time scale [33, 35]. These solutions are called *canards* and they are the basis for EADs, as demonstrated in [21].

### Canards explain the emergence and number of EADs

Many features of the slow flow persist in the flow of the full system of equations provided there is sufficient timescale separation between fast and slow variables. Theoretical justification for this persistence is provided by Fenichel theory [36, 37]. Specifically, Fenichel theory guarantees that the attracting and saddle-type sheets of the critical manifold, outside the vicinity of the fold curve, perturb smoothly to nearby *slow manifolds* under the flow of the full system, with their local attraction properties perturbing smoothly as well. In turn, the (slow) flow on these sheets is a smooth perturbation of the slow subsystem flow.

Near the folded node, the relationship between the slow subsystem flow and that of the full system is more intricate, and is described by canard theory [33–35, 38]. In particular, canard theory holds that in the neighborhood of the folded node, under the full system flow, the attracting and saddle-type sheets perturb to slow manifolds that (approximately) twist around the weak eigendirection of the folded node [33, 39]. This twisting allows the slow manifolds to be partitioned into rotational sectors, each of which oscillates around the weak eigendirection of the folded node a fixed number of times. The boundaries between different rotational sectors are curves called *maximal canards*. The first maximal canard, the boundary between the rotational sector that does not oscillate near the folded node (the left half of the upper attracting sheet) and the sector that oscillates once, is called the *primary maximal canard*.

Maximal canards have been shown to be objects of key importance in determining whether, and what kinds of potentially erratic, EAD rhythms are evoked in low-dimensional variants of the Luo-Rudy model in response to changes in ion channel expression and chemical composition of the cellular environment [21, 24, 25]. The primary maximal canard (*γ*_0_) is the perturbed analog of the slow subsystem singular strong canard ($\gamma_{0}^{0}$) and is, therefore, the boundary between standard action potentials—to its left—and those that exhibit EADs or repolarization failure—to its right. A solution that enters the rotational sector between the primary maximal canard, *γ*_0_, and the maximal canard, *γ*_1_, exhibits one canard-induced EAD; a solution that enters the rotational sector between maximal canards *γ*_1_ and *γ*_2_ exhibits two canard-induced EADs; so, in general, a solution that enters the rotational sector between *γ*_*n*_ and *γ*_*n*+1_ exhibits *n* canard-induced EADs.

**Fig 7:**
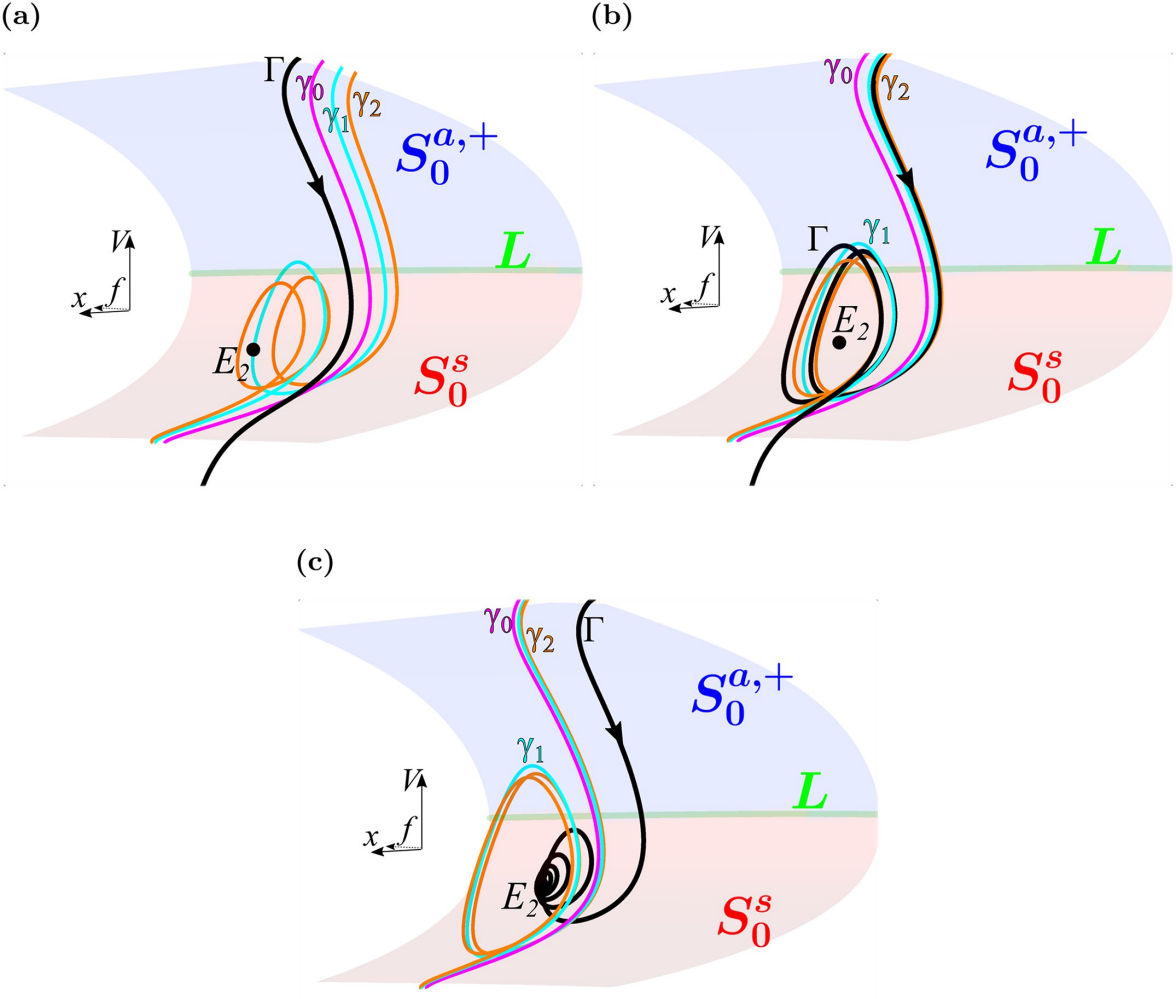
Maximal canard locations in (*f*, *x*, *V*) phase space mediate the transition from standard action potentials to repolarization failure and determine EAD number under symmetric *I*_Ca-L_ window region enlargement. (**a**) Local phase space for marker 7a in the “No EADs” region of Fig 5 (Δ*V*_1/2_(*d*_∞_) = -Δ*V*_1/2_(*f*_∞_) = -1.83 mV). The pulse-induced solution segment, Γ (black), lies to the left of the primary maximal canard, *γ*_0_ (magenta), and does not exhibit EADs. (**b**) Local phase space of marker 7b in the 2 EAD band of the “EADs” region of Fig 5 (Δ*V*_1/2_(*d*_∞_) = -Δ*V*_1/2_(*f*_∞_) = -2.08 mV). The solution segment lies within the rotational sector between maximal canards *γ*_1_ (cyan) and *γ*_2_ (orange) and exhibits two EADs. (**c**) Local phase space for marker 7c in the “Repolarization Failure” region of Fig 5 (Δ*V*_1/2_(*d*_∞_) = -Δ*V*_1/2_(*f*_∞_) = -2.33 mV). The solution segment spirals toward stable equilibrium *E*_2_, failing to return to rest. Attracting ($S_{0}^{a,+}$, blue) and saddle-type ($S_{0}^{s}$, red) sheets of the critical manifold meet at the fold curve, *L* (green). Parameter values used are listed in each panel.

Fig 7 shows key structures in phase space for responses that exhibit no EADS (Fig 7a), EADs (Fig 7b), and repolarization failure (Fig 7c). Parameter values for these behaviors are marked with ⋄ in Fig 5 labeled 7a, 7b, and 7c. Each panel shows the critical manifold and its stability properties along with the first three maximal canards (*γ*_0_, magenta; *γ*_1_, cyan; *γ*_2_, orange), computed using numerical continuation and bifurcation software AUTO [40] and methods developed in [41] which are described for this system in [21]. Also superimposed are portions of the solution segment of the full system (Γ, black) following an impulse-producing stimulus.

In Fig 7a, the solution segment (Γ, black) evolves closely along the critical manifold, and since it lies to the left of the primary maximal canard it does not exhibit EADs. Instead, it returns to the repolarized rest state to complete the action potential. However, the close proximity of Γ to *γ*_0_ extends the duration of the plateau phase of the action potential evident in the orange traces of Figs 3b and 4b. We note that the equilibrium, *E*_2_, is unstable for this choice of parameters (Δ*V*_1/2_(*d*_∞_) = -Δ*V*_1/2_(*f*_∞_) = -1.83 mV).

A solution segment with two EADs is shown in Fig 7b (red). The solution segment (Γ, black) lies to the right of *γ*_0_ (magenta) and between *γ*_1_ (cyan) and *γ*_2_ (orange), so that two small oscillations are produced, as predicted by canard theory. The equilibrium, *E*_2_, is stable for this parameter set (Δ*V*_1/2_(*d*_∞_) = -Δ*V*_1/2_(*f*_∞_) = -2.08 mV), but Γ simply does not enter its basin of attraction. However, *E*_2_ possesses a pair of complex conjugate eigenvalues (λ ± *ωi*) which, in the vicinity of *E*_2_, predict an oscillatory period (2*π*/*ω*) of ≈ 340 ms. The duration of the first and second EADs are ≈ 386 ms and ≈ 340 ms, respectively.

Fig 7c shows a case in which there is repolarization failure since the trajectory enters the basin of attraction of *E*_2_ and remains depolarized. The spiraling reflects the fact that *E*_2_ is a stable spiral equilibrium of the full system.

This analysis suggests that the responses of the model cell to window-enlarging manipulations are determined by how the manipulations affect the maximal canards in phase space. Pathological oscillatory dynamics are brought about by manipulations that translate the maximal canards leftward (in the increasing *x*-coordinate direction) relative to the solution trajectory, so that the solution trajectory enters the funnel region to the right of the primary maximal canard. Enlargement of the *I*_Ca-L_ window region can make this happen, leading to EADs or repolarization failure.

### Why left shifts of the *I*_Ca-L_ activation curve are more effective than right shifts of the inactivation curve at evoking EADs

We have shown that maximal canards mediate the transition from standard action potentials, through EADs, to repolarization failure in phase and parameter space under symmetric window enlargement. We now examine why left-shifts in the *I*_Ca-L_ activation curve are more effective than right shifts in the inactivation curve at producing EADs and repolarization failure. This should be explainable in terms of the primary maximal canard, which is the border (in phase space) of the funnel region for EADs. What effects do equally sized shifts of the activation curve *d*_∞_(*V*) and inactivation curve *f*_∞_(*V*) have on the primary maximal canard?

**Fig 8:**
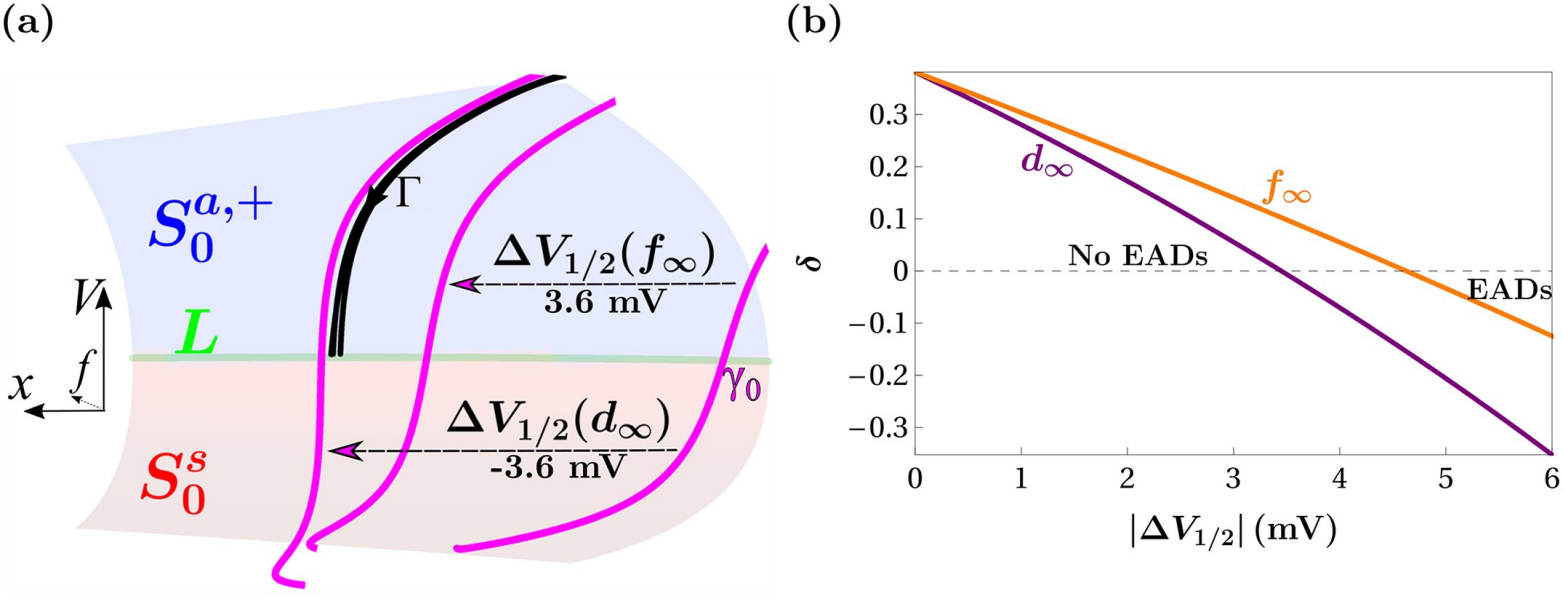
Left shifts in the Ca^2+^ channel activation curve move the primary maximal canard further than equal right shifts in the inactivation curve. (**a**) Three primary maximal canards corresponding to default (*γ*_0_, right, magenta), right-shifted *f*_∞_(*V*) (middle, magenta), and left-shifted *d*_∞_(*V*) (left, magenta) conditions are superimposed on the critical manifold of the default parameter set. Also shown is a portion of the trajectory during the plateau phase of an action potential (Γ, black) for each condition. These three trajectory segments are almost identical, but the one corresponding to left-shifted *d*_∞_(*V*) enters the funnel and will subsequently exhibit EADs. (**b**) The distance, *δ*, between Γ and *γ*_0_ declines faster with left shifts in *d*_∞_(*V*) than with right shifts in *f*_∞_(*V*).

Fig 8a shows a phase-space view with the critical manifold and the primary maximal canard *γ*_0_ (magenta) prior to a shift in the activation/inactivation curves. When the Ca^2+^ channel activation curve is left shifted by 3.6 mV (Δ*V*_1/2_(*d*_∞_) = −3.6 mV) the primary maximal canard moves leftward in phase space, as indicated in the figure. An equal right shift in the inactivation curve (Δ*V*_1/2_(*f*_∞_) = 3.6 mV) also moves *γ*_0_ leftward, but not as far. The figure also includes a portion of the trajectory during the action potential plateau (Γ, black) with and without a shift in either the activation or inactivation curve. It is apparent that the shift in these curves has very little effect on this portion of the trajectory (the three black segments are very close together), however with the shift in the activation curve the trajectory enters the funnel and will exhibit EADs, while with the equal shift of the inactivation curve it will not. Thus, the reason that EADs are facilitated more by left shifts in the activation curve than right shifts in the inactivation curve is that the primary maximal canard is affected more by the former maneuver than the latter.

To make these arguments more precise, in Fig 8b we introduce a quantity, *δ*, that measures the signed distance between a point on the pulsed solution Γ (that also lies on the slow manifold corresponding to $S_{0}^{a,+}$) and the primary maximal canard, *γ*_0_, as a function of the shift magnitude, |Δ*V*_1/2_|, in either *d*_∞_(*V*) (purple curve) or *f*_∞_(*V*) (orange curve). Positive values of *δ* indicate that Γ lies to the left of *γ*_0_ (no EADs), while negative values of *δ* indicate that Γ lies to the right of *γ*_0_ (EADs or repolarization failure). Zeros of *δ* indicate that Γ coincides with *γ*_0_ and is the boundary between action potentials with and without EADs; zeros correspond to points on the green boundary curve in Fig 5. The locations of the zeros of *δ* are unaffected by the point on Γ (that coincides with the slow manifold) from which the measurements are made.

In agreement with Fig 8a (and Fig 5), we find that *δ* decreases more rapidly for left shifts in *d*_∞_(*V*) (purple curve) than for right shifts in *f*_∞_(*V*) (orange curve), corresponding to more rapid leftward movement of *γ*_0_ toward Γ under left-shifts in *d*_∞_(*V*). As a result, *δ* crosses zero (near |Δ*V*_1/2_| ≈ 3.45 mV) as |Δ*V*_1/2_| increases toward 3.6 mV for *d*_∞_(*V*), while *δ* remains greater than 0 over the same range of |Δ*V*_1/2_| for *f*_∞_(*V*).

### A left shift in the *I*_Ca-L_ activation curve narrows the parameter range for EADs by constricting the maximal canards

**Fig 9:**
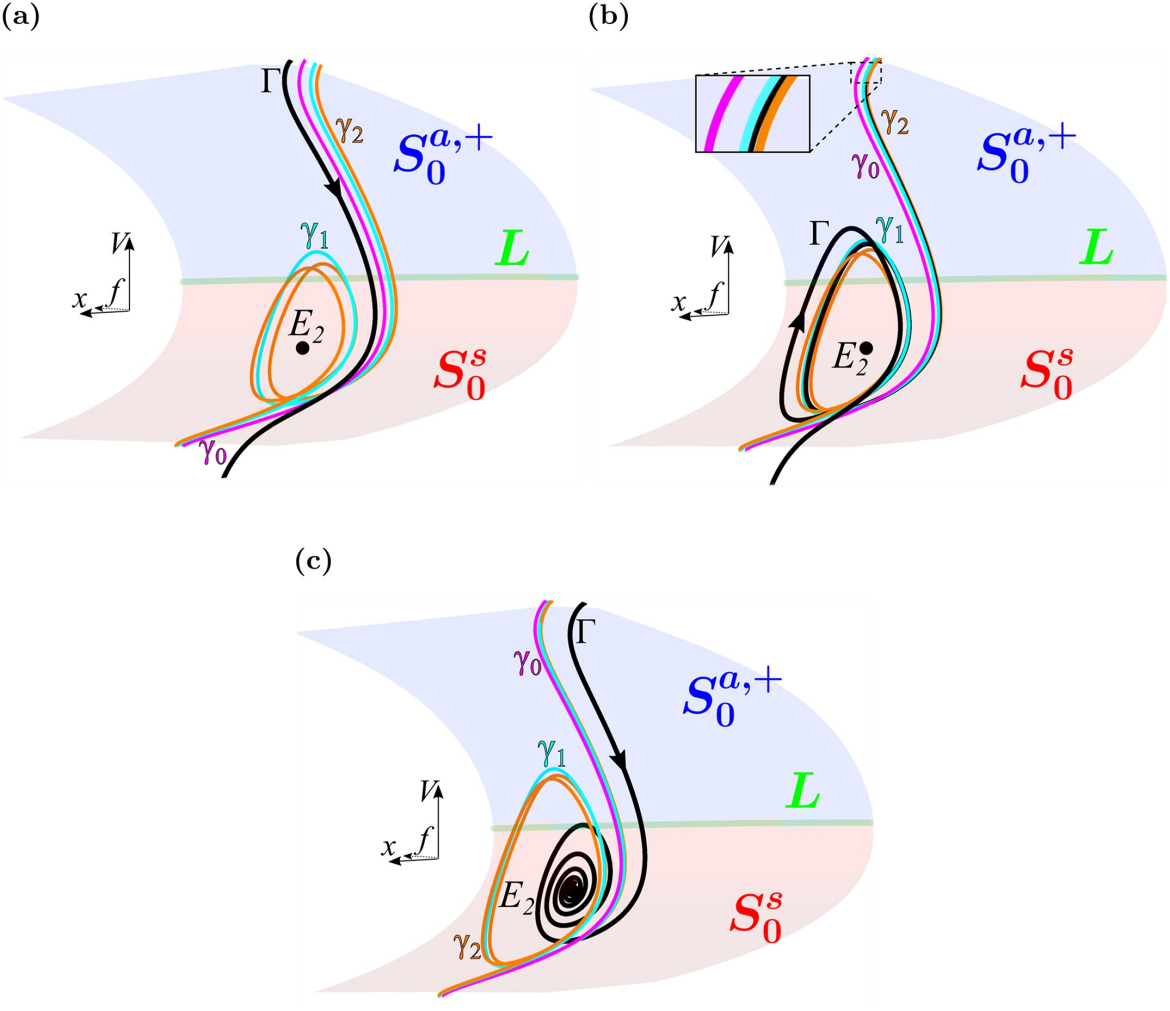
Maximal canards shift leftward and constrict with increasing left shifts in *d*_∞_(*V*). (**a**) At a value of Δ*V*_1/2_(*d*_∞_) (= -3.35 mV) where no EADs are produced (corresponding to ⋄ marker 9a in Fig 5) the trajectory lies outside the funnel region for EADs. (**b**) With a somewhat greater shift in *d*_∞_(*V*) (Δ*V*_1/2_(*d*_∞_) = -3.6 mV), corresponding to the ⋄ marker 9b in Fig 5, the trajectory enters the region between *γ*_1_ (cyan) and *γ*_2_ (orange) and two EADs are produced. The maximal canards have shifted leftward and are closer together than in the first panel. (**c**) With an even greater left-shift in *d*_∞_(*V*) (Δ*V*_1/2_(*d*_∞_) = -3.85 mV) the trajectory is attracted to equilibrium *E*_2_ and there is repolarization failure. With this greater shift the maximal canards are even more constricted.

One peculiar observation from Fig 5 is that the EAD sector (in red) is narrow at the bottom and wider at the top. This means that with a large left-shift in *d*_∞_(*V*) the range of right-shifts in *f*_∞_(*V*) that can produce EADs becomes smaller. Why is this? To address this question, we examine the maximal canards in phase space for three values of Δ*V*_1/2_(*d*_∞_) (⋄ markers in Fig 5). The first panel of Fig 9 shows the situation when the left-shift in *d*_∞_(*V*) is not large enough to evoke EADs. In this case, the trajectory segment lies to the left of *γ*_0_ and thus outside the funnel. In the second panel, with a larger left shift, the trajectory lies between *γ*_1_ (cyan) and *γ*_2_ (orange), so two EADs are produced. In the third panel, the trajectory spirals into the equilibrium *E*_2_ and there is repolarization failure.

What is important to observe in Fig 9 is that the spacing between the maximal canards gets smaller for large left shifts in *d*_∞_(*V*). Thus, there is a constriction of the region in phase space where EADs, rather than repolarization failure, are evoked. Constriction of the phase space region where EADs are evoked also occurs with right shifts in *f*_∞_(*V*), but the rate and severity are less pronounced. This too corroborates a prediction from canard theory. In the singular limit, the ratio of the eigenvalues of the folded node, *μ* ≔ λ_*w*_/λ_*s*_ < 1, can be used to estimate how densely the secondary maximal canards (*γ*_1_, *γ*_2_, etc.) accumulate near the primary maximal canard (*γ*_0_) in the full system flow (see Propositions 3.5 and 3.6 of [38]). We find that *μ* decreases more rapidly for left shifts in *d*_∞_(*V*) than for right shifts in *f*_∞_(*V*), which predicts that the maximal canards will accumulate more densely on the primary maximal canard under left shifts *d*_∞_(*V*), as we observe. It is for this reason that the EAD region in Fig 5 is narrow at the bottom and wider at the top.

### Decreasing the size of the window region can compensate for pathological conditions that promote EADs

While broadening the *I*_Ca-L_ window can lead to pathological electrical rhythms, it is also plausible that pathological conditions can be compensated for by narrowing the window. In vitro experiments with isolated cardiomyocytes and cardiac tissue have shown that simulating hypokalemia by reducing the extracellular K^+^ concentration in the bath reliably elicits EADs [8, 11, 42, 43]. In [21], we showed that simulating hypokalemia (by reducing the parameter [K^+^]_o_) in the model (1) also elicits EADs, due to a canard mechanism similar to that described above. In [14] it was shown that narrowing the *I*_Ca-L_ window in dynamic clamp experiments can overcome the effects of low extracellular K^+^ and eliminate the EADs. Can this also be explained by the model?

**Fig 10:**
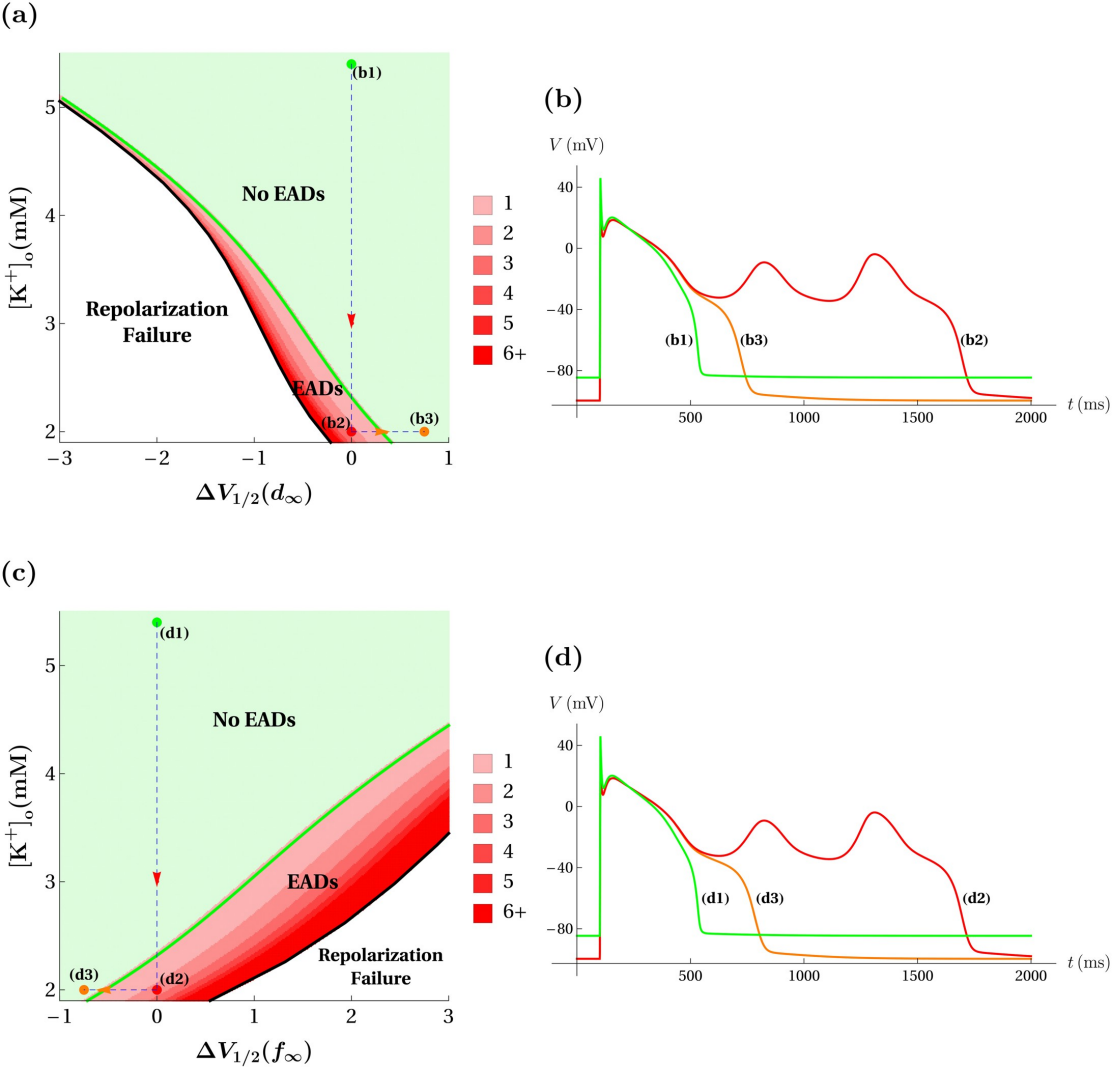
Narrowing the Ca^2+^ current window by shifting *d*_∞_(*V*) or *f*_∞_(*V*) eliminates hypokalemia-induced EADs in the model. (**a**) Model responses in the (Δ*V*_1/2_(*d*_∞_), [K^+^]_o_) parameter plane. The green marker (b1) denotes the default condition, the red marker (b2) denotes the hypokalemia condition, and the orange marker (b3) denotes the *d*_∞_(*V*)-shifted hypokalemia condition. (**b**) Voltage time courses for the color-matched markers (b1), (b2), and (b3) of panel (a). (**c**) Model responses in the (Δ*V*_1/2_(*f*_∞_), [K^+^]_o_) parameter plane. The green marker (d1) denotes the default condition, the red marker (d2) denotes the hypokalemia condition, and the orange marker (d3) denotes the *f*_∞_(*V*)-shifted hypokalemia condition. (**d**) Voltage time courses for the color-matched markers (d1), (d2), and (d3) of panel (c).

To investigate, we reduced the extracellular K^+^ concentration parameter [K^+^]_o_ over a range of values, which has the effect of increasing the K^+^ Nernst potentials, *V*_K_ and *V*_K1_, while decreasing the maximal conductances, *g*_K_ and *g*_K1_. We also translated the Ca^2+^ activation curve *d*_∞_(*V*) over a range of values so as to evaluate the combined effects of these maneuvers. The top panels of Fig 10 show the result. The green marker labelled b1 (Fig 10a) shows that with the default [K^+^]_o_(= 5.4 mM) and no shift in *d*_∞_(*V*) a standard action potential is produced (Fig 10b). In fact, for any shift in *d*_∞_(*V*) a standard action potential is produced. For lower values of [K^+^]_o_ (simulating hypokalemia), EADs become possible if *d*_∞_(*V*) is left shifted. For a sufficiently low value of [K^+^]_o_, EADs occur even with no left-shift in *d*_∞_(*V*). This is the case with [K^+^]_o_ = 2.0 mM shown with the red marker labelled b2 in Fig 10a. With this parameter combination two EADs are produced, greatly extending the duration of the action potential (Fig 10b). However, if *d*_∞_(*V*) is then right shifted (Δ*V*_1/2_(*d*_∞_) = 0.75 mV), to the orange point labelled b3 (Fig 10a) the EADs are eliminated, yielding an action potential of almost-normal duration (Fig 10b). Thus, right shifts in *d*_∞_(*V*) can eliminate the EADs brought about by hypokalemia in model simulations.

Fig 10c and 10d show a similar scenario, but in this case left-shifts in *f*_∞_(*V*) are used to narrow the Ca^2+^ current window. Starting from the default value of [K^+^]_o_ and with no shift (green point d1), simulated hypokalemia brings the system into the EAD region (red point d2). Applying a left-shift to *f*_∞_(*V*) of Δ*V*_1/2_(*f*_∞_) = −0.75 mV eliminates the EADs (orange point d3). Thus, both window-narrowing maneuvers produce the desired result of eliminating hypokalemia-induced EADs. Because the EAD region is smaller in Fig 10a than in Fig 10c, it would generally be more successful in the model to eliminate EADs in conditions of hypokalemia with shifts in *d*_∞_(*V*) than with shifts in *f*_∞_(*V*), as observed experimentally in [14].

Given the importance of excess *I*_Ca-L_ in the production of EADs, it is not surprising that when the Ca^2+^ current conductance was increased during dynamic clamp experiments there was an increase in EAD production and repolarization failure. These effects were eliminated when the *I*_Ca-L_ window was symmetrically narrowed [15]. We demonstrate that the model (1) recapitulates both the increase in propensity of repolarization failure with an increase in *g*_Ca_ and the rescue of a standard action potential with appropriate symmetric narrowing of the *I*_Ca-L_ window.

**Fig 11:**
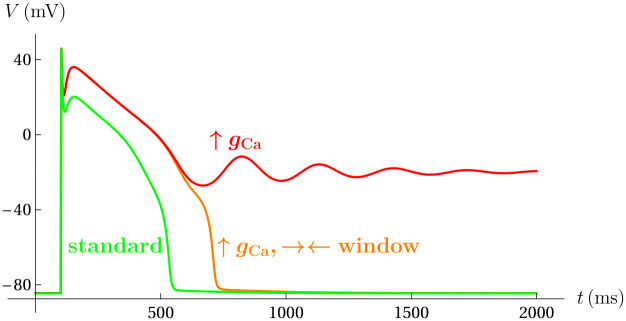
Symmetric narrowing of the model window region abolishes *I*_Ca-L_ amplitude-induced repolarization failure. Repolarization failure is promoted by increasing the conductance of the *I*_Ca-L_ current (red). Narrowing the window recovers the action potential response (orange). Green: (*g*_Ca_, Δ*V*_1/2_(*d*_∞_), Δ*V*_1/2_(*f*_∞_)) = (0.112, 0, 0); orange: (*g*_Ca_, Δ*V*_1/2_(*d*_∞_), Δ*V*_1/2_(*f*_∞_)) = (0.18, 0, 0); red: (*g*_Ca_, Δ*V*_1/2_(*d*_∞_), Δ*V*_1/2_(*f*_∞_)) = (0.18, 1, -1).

In Fig 11, the conversion of an action potential (green) to repolarization failure (red) in response to an increase in *g*_Ca_ (to 0.18 mS/cm^2^) is illustrated. By symmetrically narrowing the *I*_Ca-L_ window with Δ*V*_1/2_(*d*_∞_) = 1 mV and Δ*V*_1/2_(*f*_∞_) = −1 mV, there is recovery of an action potential response to the stimulus. In a physiological setting, this and the previous result suggest that dynamic regulation of the *I*_Ca-L_ window can be very effective at overcoming pathological conditions leading to EADs and repolarization failure.

### Changes in Ca^2+^ channel time constants are predicted to eliminate hypokalemia-induced EADs

We have shown that the model reproduces many of the experimental results obtained with dynamic clamp in [14] and [15]. We have also shown that the EADs induced under these manipulations can be explained mathematically as canard-induced oscillations. We now extend our analysis by using the model to make predictions about the anti-arrhythmic effects of altering kinetic properties of the Ca^2+^ current. Specifically, we examine model responses to changes in the time constants of *I*_Ca-L_ activation, *τ*_*d*_(*V*), and inactivation, *τ*_*f*_(*V*), under simulated hypokalemia.

To examine the effects of changing Ca^2+^ current time constants we multiply the voltage-dependent timescale functions by scaling parameters, *α* and *β*. Then the activation and inactivation variables change in time according to:

$$\begin{array}{c} \begin{array}{cc} \frac{dd}{dt} & =\frac{d_{\infty }\left(V\right)-d}{\alpha \tau_{d}\left(V\right)}\\ \frac{df}{dt} & =\frac{f_{\infty }\left(V\right)-f}{\beta \tau_{f}\left(V\right)} \end{array} \end{array}$$ (5)

Values of a scaling parameter larger than 1 make the corresponding time constant larger and thus slow the rate of adjustment of the corresponding gating variable to the variations in *V*; values of a scaling parameter less than 1 hasten this adjustment.

**Fig 12:**
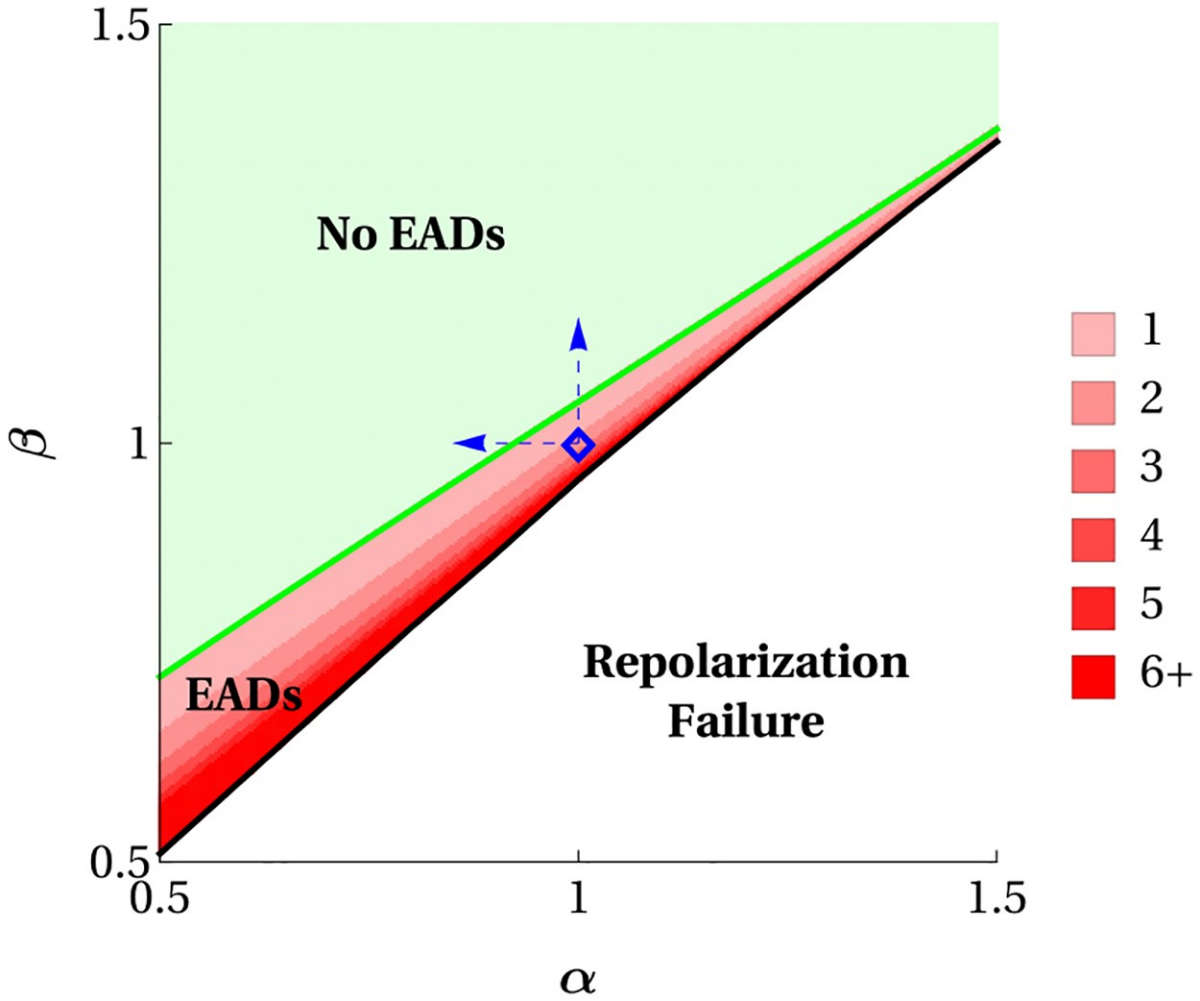
Model responses to variation in scaling parameters of Ca^2+^ channel activation (*α*) and inactivation (*β*) timescales under simulated hypokalemia. The blue ⋄ marker denotes the hypokalemia condition of Fig 10 and the blue dashed arrows highlight two separate dynamic clamp manipulations predicted to eliminate hypokalemia-induced EADs.

The model responses to independent variation in *α* and *β* are shown in Fig 12. For reference, the blue ⋄ marker in the two EADs band of the red “EADs” region of Fig 12 denotes the baseline hypokalemia condition ([K^+^]_o_ = 2.0 mM) in the absence of time constant manipulations. Two dashed blue arrows, one pointing leftward toward decreases in *α* alone and the other pointing upward toward increases in *β* alone, show separate manipulations that predict the elimination of hypokalemia-induced EADs. The EAD-eliminating decreases in *α* correspond to more rapid activation of *I*_Ca-L_ in response to a depolarizing stimulus while the EAD-eliminating increases in *β* correspond to delayed inactivation of *I*_Ca-L_ during an actio potential. These results seem counterintuitive, since the first manipulation makes *I*_Ca-L_ turn on faster and the second makes it turn off slower in response to a stimulus. Why would manipulations that are expected to prolong the influence of a depolarizing current shorten action potentials and reduce the likelihood of EADs?

The answer again lies in the fast-slow analysis and, in particular, the location of the primary maximal canard *γ*_0_ with respect to the location of the pulsed solution Γ in phase space. As we discussed earlier, and showed in detail in [21], the primary maximal canard moves far to the left of the singular strong canard as parameters are changed that move the system away from the singular limit. When the time constant for *d* is decreased or that for *f* is increased, this has the effect of further separating the timescales of fast and slow variables. That is, it moves the system closer to the singular limit. As a result, *γ*_0_ moves rightward towards $\gamma_{0}^{0}$, and in the process crosses Γ, so that Γ now falls outside of the funnel region so no EADs are produced.

## Discussion

Recent studies using the dynamic clamp experimental technique have demonstrated that the *I*_Ca-L_ window region, the voltage range over which the activation and inactivation curves overlap, plays an important role in regulating myocyte electrical rhythms [14, 15]. They showed that EADs and repolarization failure are facilitated by window broadening, and that conditions promoting these pathological electrical behaviors could be overcome by narrowing the window. In this manuscript we demonstrated that a 4-dimensional variant of the Luo-Rudy I model [30] can reproduce and explain these findings. The low dimensionality of the model allowed us to perform a fast-slow analysis, enabling our ability to view the EADs as canard-induced phenomena. In particular, we showed that the EADs produced under changes in the size of the window region are canard-induced oscillations and that the canards can be used to explain many of the effects of different manipulations reported in [14] and [15]. With this technique, we demonstrated that it is even possible to explain why a particular number of EADs is elicited under a given parameter regime.

The size of the *I*_Ca-L_ window region is determined by the configuration of both the activation and inactivation curves. Hence, enlarging the window region can be accomplished by shifts in either or both curves. Dynamic clamp experiments in [15] showed that both simultaneous and independent window-enlarging shifts in the activation and inactivation curves are capable of producing EADs. Figs 3 and 4 replicate these findings. But why does enlarging the window region lead to EADs and repolarization failure? The biophysical explanation is that the enlarged window allows for sustained activation of the current, and indeed this is true. But why does the voltage oscillate to give EADs rather than just give an extended plateau? This is best explained mathematically. In the model, there is a twisted funnel region in phase space whose position changes with the configuration of the *I*_Ca-L_ window region. Smaller window regions keep this funnel away from where solutions are injected following a depolarizing pulse so that solutions do not experience twist-induced oscillations, while larger window regions move the funnel toward or across where solutions are injected which leads to EAD oscillations. Hence, the pro-arrhythmic potency of one manipulation over another, as is shown in Fig 4 for left shifts in the activation curve versus right shifts in the inactivation curve, can be explained by tracking the respective movements of the curve (primary maximal canard) that bounds the funnel region for oscillations (Fig 7).

Why is it useful to cast the window region in terms of canards and twisted slow manifolds? The reason is predictability. Knowledge of the size of the Ca^2+^ current window is only useful within the context of other biophysical parameters. We demonstrated this by showing that the window size for EADs is highly dependent on the external K^+^ concentration (Fig 10a and 10c). Also, changing the number of Ca^2+^ channels in the cell’s membrane affects whether the window region is appropriate for EADs or repolarization block, as we demonstrated in Fig 11. So knowing the size of the window region is insufficient for knowing whether EADs or repolarization block will occur. Knowing the geometric structure of the model, in particular the phase space locations of the maximal canards, provides much more precise information and allows us to interpret in a straight-forward way what happens when *d*_∞_(*V*) or *f*_∞_(*V*) are shifted and the window region modified. It also allows us to predict which changes in biophysical parameters (and their magnitudes) elicit EADs or repolarization block.

The predictive capacity of the fast-slow analysis was also demonstrated by our finding that increasing the rate of Ca^2+^ channel activation or decreasing the rate of inactivation under hypokalemia conditions can eliminate EADs (Fig 12). This prediction emerges naturally from the analysis, but is not at all obvious from biophysical arguments alone. While the effects of time constant manipulations were not considered in the two dynamic clamp studies that are the focus of this work [14, 15], another study [44] did test the effects of such manipulations, but only in the case of H_2_O_2_-induced EADs. The latter study found that manipulating the time constants of Ca^2+^ channel activation and inactivation had small effects on existent EADs, although the direction of the effects are in agreement with the predictions made here for small-magnitude manipulations. The computer-generated Ca^2+^ current used in [44] contains a voltage-dependent inactivation curve with incomplete inactivation, which produces a persistent “pedestal” current. The major finding of [44] was that a larger pedestal current (reduced inactivation) promoted both H_2_O_2_- and hypokalemia-induced EADs. We found that the addition of such a pedestal current in the present model led to an increase in the number of EADs induced under hypokalemia conditions.

There have been many computational models of cardiac APs developed since the original Luo-Rudy model [30]. Most of these models contain more detailed descriptions of transmembrane ionic currents and intracellular ion handling as experiments have continued to uncover important features of the intracellular and membrane biophysics of cardiac cells. For this reason, these models are often high dimensional. For example, one well-regarded model contains more than 40 dynamic variables [29]. Many of these models have been shown to produce EADs under parameter regimes that represent the same kinds of manipulations tested in the current work. In addition, some of these models can also produce EADs through biophysical mechanisms that are not present in the Luo-Rudy model, such as maladaptive calcium-induced calcium release [17, 18, 45] or reactivation of the late Na^+^ current [46, 47]. The central role played by canards in the present minimal model, and others, highlights the plausibility for such a central role for canards in these more complex models. It is quite possible that EADs in a high-dimensional model are due to a twisted slow manifold, even though demonstrating that would be very difficult due to the high dimensionality. It is also possible that canards are responsible for the EADs generated by maladaptive CICR. Indeed, we speculate that a single dynamical mechanism—canards—may be responsible for many instances of EADs generated through either a purely electrical mechanism or through CICR.

Cellular EADs have been linked to tissue-level arrhythmias, but the precise relationship between the prolongation of cellular action potential duration (APD) and the lethality of tissue level arrhythmia is not well understood. For instance, *Torsades de pointes*, a tissue-level tachycardic arrhythmia caused by cellular APD prolongation (observed as long QT syndromes) can either occur as a transient tissue behavior that spontaneously self-extinguishes or a sustained dysrhythmia that devolves into full ventricular fibrillation and heart failure. The canard mechanism, shown in this work to underlie cellular EADs, provides a new potential line of inquiry for investigating the propagation and synchronization of cellular rhythms at the tissue-level.
